# Dichlorido[*N*′-(3,5-dichloro-2-hydroxy­benzyl­idene)pyridine-4-carbohydrazide-κ*N*](1,10-phenanthroline-κ^2^ *N*,*N*′)cobalt(II) methanol monosolvate

**DOI:** 10.1107/S1600536809047680

**Published:** 2009-11-18

**Authors:** Yuan Wang, Zheng Liu, Baoyu Liu

**Affiliations:** a Key Laboratory of Nonferrous Metal Materials and Processing Technology, Department of Material and Chemical Engineering, Guilin University of Technology, Ministry of Education, Guilin 541004, People’s Republic of China, and College of Chemical and Biological Engineering, Guilin University of Technology, Guilin 541004, People’s Republic of China

## Abstract

In the title compound, [CoCl_2_(C_13_H_9_Cl_2_N_3_O_2_)_2_(C_12_H_8_N_2_)]·CH_3_OH, the Co^II^ atom is octahedrally coordinated by two N atoms from the pyridyl rings of the tridentate *N*′-(3,5-dichloro-2-hydroxy­benzyl­idene)pyridine-4-carbohydrazide (H_2_ *L*) ligand, two N atoms from the 1,10-phenanthroline ligand and two chloride ions. The acyl­hydrazone groups are not involved into the coordination of the metal ion. In the crystal packing an extended three-dimensional network formed by N—H⋯Cl, N—H⋯O, O—H⋯N, O—H⋯N and O—H⋯Cl hydrogen bonds is observed.

## Related literature

For acyl­hydrazone complexes containing heteroatoms, see: Adams *et al.* (2000 ▶); Kuriakose *et al.* (2007 ▶); Lobana *et al.* (2006 ▶); Mujeebur Rahman *et al.* (2005 ▶). For a related structure, see: Armstrong *et al.* (2003 ▶).
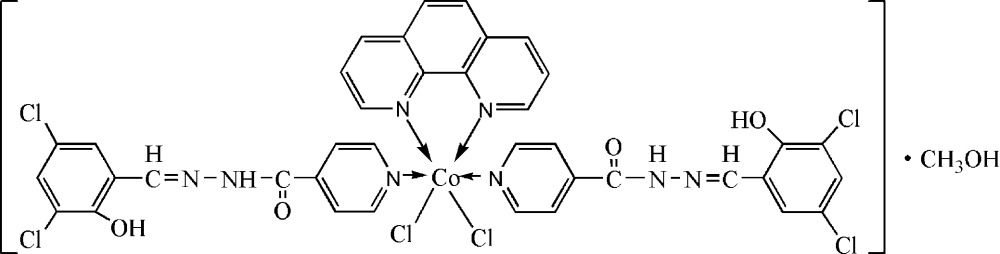


## Experimental

### 

#### Crystal data

[CoCl_2_(C_13_H_9_Cl_2_N_3_O_2_)_2_(C_12_H_8_N_2_)]·CH_4_O
*M* *_r_* = 962.34
Orthorhombic, 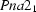

*a* = 20.797 (3) Å
*b* = 14.1641 (16) Å
*c* = 13.7952 (10) Å
*V* = 4063.7 (7) Å^3^
*Z* = 4
Mo *K*α radiation
μ = 0.87 mm^−1^
*T* = 298 K
0.32 × 0.23 × 0.22 mm

#### Data collection

Bruker SMART CCD area-detector diffractometer
Absorption correction: multi-scan (*SADABS*; Sheldrick, 1996 ▶) *T*_min_ = 0.768, *T*_max_ = 0.831
17533 measured reflections
7003 independent reflections
3870 reflections with *I* > 2σ(*I*)
*R*_int_ = 0.052

#### Refinement

*R*[*F* ^2^ > 2σ(*F* ^2^)] = 0.056
*wR*(*F* ^2^) = 0.170
*S* = 1.03
7003 reflections
532 parameters
1 restraint
H-atom parameters constrained
Δρ_max_ = 0.51 e Å^−3^
Δρ_min_ = −0.57 e Å^−3^
Absolute structure: Flack (1983 ▶), 3265 Friedel pairs
Flack parameter: 0.50 (3)

### 

Data collection: *SMART* (Bruker, 2001 ▶); cell refinement: *SAINT* (Bruker, 2001 ▶); data reduction: *SAINT*; program(s) used to solve structure: *SHELXS97* (Sheldrick, 2008 ▶); program(s) used to refine structure: *SHELXL97* (Sheldrick, 2008 ▶); molecular graphics: *SHELXTL* (Sheldrick, 2008 ▶); software used to prepare material for publication: *SHELXTL*.

## Supplementary Material

Crystal structure: contains datablocks global, I. DOI: 10.1107/S1600536809047680/vm2009sup1.cif

Structure factors: contains datablocks I. DOI: 10.1107/S1600536809047680/vm2009Isup2.hkl

Additional supplementary materials:  crystallographic information; 3D view; checkCIF report

## Figures and Tables

**Table 1:** Selected geometric parameters (Å, °)

Co1—N8	2.170 (7)
Co1—N7	2.170 (7)
Co1—N1	2.217 (6)
Co1—N4	2.235 (7)
Co1—Cl1	2.401 (2)
Co1—Cl2	2.419 (2)
N2—N3	1.368 (8)
N5—N6	1.389 (9)

**Table 2:** Hydrogen-bond geometry (Å, °)

*D*—H⋯*A*	*D*—H	H⋯*A*	*D*⋯*A*	*D*—H⋯*A*
N2—H2⋯Cl1^i^	0.86	2.56	3.280 (7)	142
N5—H5⋯O5	0.86	1.91	2.739 (11)	162
O2—H2*A*⋯N3	0.82	1.85	2.562 (8)	145
O4—H4⋯N6	0.82	1.88	2.592 (9)	145
O5—H5*A*⋯Cl2^ii^	0.82	2.24	3.052 (9)	171

Symmetry codes: (i) 
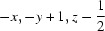
; (ii) 
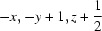
.

**Fig. 1.**
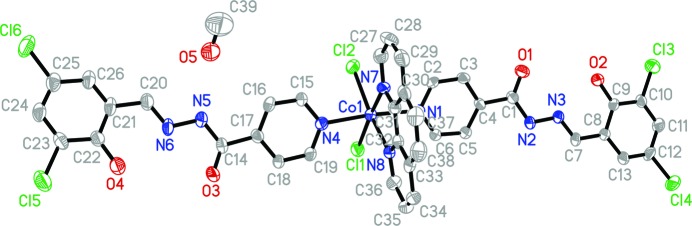
The asymmetric unit of (I), showing 30% probability displacement ellipsoids. Carbon-bound H atoms have been omitted.

**Fig. 2.**
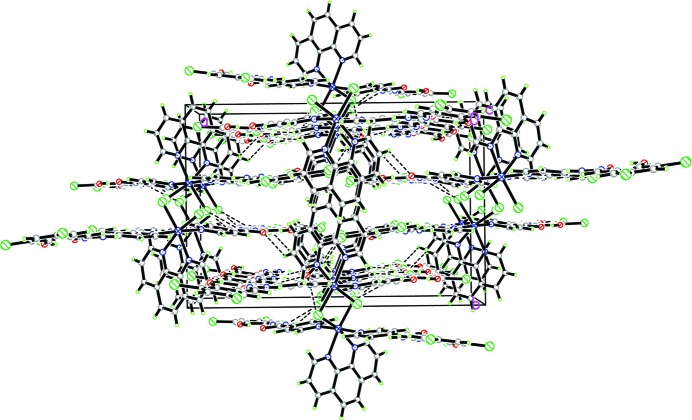
Three-dimensional network in crystal packing of (I), broken lines show N–H···Cl, N–H···O, O–H···N, O–H···N and O–H···Cl hydrogen bonds.

## supplementary crystallographic information

### Comment

In the field of coordination chemistry, continuing interest in the acylhydrazones transition metal complexes stems from their analytical, catalytic chemistry and as models for metalloenzymes. Acylhydrazone ligands can act as bidentate, tridentate or tetradentate ligands depending on the nature of heterocyclic ring substituents attached to the hydrazone unit.

In (I), there is methanol solvate molecule, and the Co^II^ atom is coordinated by two N atoms from pyridyls of H_2_L and two N atoms from 1,10-phenanthroline and two Cl ions, which form a slightly distorted tetragonal-dipyramid geometry (Fig. 1). From the bond lengths of (I), we can find the N atoms from 1,10-phenanthroline possess stronger coordinating capability compared to the pyridyls. The acylhydrazone of (I) is a kind of polydentate ligand which contains three heteroatoms. However, the acylhydrazone groups are not involved in the coordination. On the other hand, this phenomenon illustrates the pyridyl N atom of H_2_L has a stronger coordinating capability than the acylhydrazone group. Also in the structure of 2-pyridinecarbaldehyde isonicotinoylhydrazone and manganese chloride at 2:1 mole ratio no coordination of the acylhydrazone groups with the metal ion was observed (Armstrong *et al.*, 2003). The three-dimensional network through N–H···Cl, N–H···O, O–H···N, O–H···N and O–H···Cl hydrogen bonds in the packing of (I) is shown in Figure 2.

### Experimental

An EtOH solution (30 ml) of 3,5-Dichlorosalicylaldehyde (10 mmol) was added dropwise to the EtOH solution (20 ml) of 4-Pyridinecarboxylic acid hydrazide (10 mmol) with stirring at *ca* 75\ % C for 3 h. The white precipitates was removed by filtration and recrystallized from EtOH solution. Then a mixture of the ligand (0.5 mmol) and cobalt chloride (0.5 mmol) in MeOH (35 ml) was stirred at *ca* 65\ % C for 45 min to give the red precipitates. Add 10 ml MeOH solution of 1,10-phenanthroline (0.5 mmol) to the mixture and stirred for 1.5 h. The red precipitate decreased gradually. Then the mixture was filtrated and ether evaporated slowly to afford almost quantitatively red crystals of mononuclear complex at ambient temperature after several days.

### Crystal data

**Table:**

[CoCl_2_(C_13_H_9_Cl_2_N_3_O_2_)_2_(C_12_H_8_N_2_)]·CH_4_O	*F*(000) = 1956
*M**_r_* = 962.34	*D*_x_ = 1.573 Mg m^−^^3^
Orthorhombic, *P**n**a*2_1_	Mo *K*α radiation, λ = 0.71073 Å
Hall symbol: P 2c -2n	Cell parameters from 3420 reflections
*a* = 20.797 (3) Å	θ = 2.3–25.2°
*b* = 14.1641 (16) Å	µ = 0.87 mm^−^^1^
*c* = 13.7952 (10) Å	*T* = 298 K
*V* = 4063.7 (7) Å^3^	Block, red
*Z* = 4	0.32 × 0.23 × 0.22 mm

### Data collection

**Table:**

Bruker SMART CCD area-detector diffractometer	7003 independent reflections
Radiation source: fine-focus sealed tube	3870 reflections with *I* > 2σ(*I*)
graphite	*R*_int_ = 0.052
φ and ω scans	θ_max_ = 25.0°, θ_min_ = 1.7°
Absorption correction: multi-scan (*SADABS*; Sheldrick, 1996)	*h* = −16→24
*T*_min_ = 0.768, *T*_max_ = 0.831	*k* = −16→15
17533 measured reflections	*l* = −15→16

### Refinement

**Table:**

Refinement on *F*^2^	Secondary atom site location: difference Fourier map
Least-squares matrix: full	Hydrogen site location: inferred from neighbouring sites
*R*[*F*^2^ > 2σ(*F*^2^)] = 0.056	H-atom parameters constrained
*wR*(*F*^2^) = 0.170	*w* = 1/[σ^2^(*F*_o_^2^) + (0.0742*P*)^2^ + 2.7173*P*] where *P* = (*F*_o_^2^ + 2*F*_c_^2^)/3
*S* = 1.03	(Δ/σ)_max_ = 0.001
7003 reflections	Δρ_max_ = 0.51 e Å^−^^3^
532 parameters	Δρ_min_ = −0.57 e Å^−^^3^
1 restraint	Absolute structure: Flack (1983), 3259 Friedel pairs
Primary atom site location: structure-invariant direct methods	Flack parameter: 0.50 (3)

### Special details

**Table:**

Geometry. All e.s.d.'s (except the e.s.d. in the dihedral angle between two l.s. planes) are estimated using the full covariance matrix. The cell e.s.d.'s are taken into account individually in the estimation of e.s.d.'s in distances, angles and torsion angles; correlations between e.s.d.'s in cell parameters are only used when they are defined by crystal symmetry. An approximate (isotropic) treatment of cell e.s.d.'s is used for estimating e.s.d.'s involving l.s. planes.
Refinement. Refinement of *F*^2^ against ALL reflections. The weighted *R*-factor *wR* and goodness of fit *S* are based on *F*^2^, conventional *R*-factors *R* are based on *F*, with *F* set to zero for negative *F*^2^. The threshold expression of *F*^2^ > σ(*F*^2^) is used only for calculating *R*-factors(gt) *etc*. and is not relevant to the choice of reflections for refinement. *R*-factors based on *F*^2^ are statistically about twice as large as those based on *F*, and *R*- factors based on ALL data will be even larger.

### Fractional atomic coordinates and isotropic or equivalent isotropic displacement parameters (Å^2^)

**Table:**

	*x*	*y*	*z*	*U*_iso_*/*U*_eq_	
Co1	0.00635 (5)	0.61639 (7)	0.39038 (8)	0.0510 (3)	
Cl1	0.05943 (11)	0.47788 (16)	0.32986 (17)	0.0678 (6)	
Cl2	−0.06639 (11)	0.53739 (18)	0.50061 (16)	0.0687 (6)	
Cl3	−0.44946 (12)	0.6158 (2)	−0.3164 (2)	0.0958 (9)	
Cl4	−0.26296 (14)	0.6306 (2)	−0.57949 (18)	0.1032 (10)	
Cl5	0.48041 (14)	0.6604 (3)	1.0861 (2)	0.1090 (11)	
Cl6	0.28736 (17)	0.6082 (2)	1.3338 (2)	0.1105 (11)	
N1	−0.0618 (3)	0.6114 (4)	0.2669 (5)	0.0461 (16)	
N2	−0.1804 (3)	0.6209 (4)	−0.0562 (5)	0.0560 (18)	
H2	−0.1402	0.6179	−0.0707	0.067*	
N3	−0.2272 (3)	0.6245 (4)	−0.1259 (4)	0.0526 (17)	
N4	0.0816 (3)	0.6290 (4)	0.5052 (5)	0.0532 (17)	
N5	0.2138 (4)	0.6320 (5)	0.8120 (5)	0.066 (2)	
H5	0.1740	0.6326	0.8289	0.079*	
N6	0.2631 (3)	0.6351 (5)	0.8798 (5)	0.0615 (18)	
N7	−0.0302 (3)	0.7527 (5)	0.4367 (5)	0.0557 (17)	
N8	0.0619 (3)	0.7165 (5)	0.3057 (4)	0.0503 (17)	
O1	−0.2578 (3)	0.6306 (5)	0.0585 (4)	0.0735 (19)	
O2	−0.3461 (3)	0.6239 (4)	−0.1741 (4)	0.0676 (16)	
H2A	−0.3156	0.6264	−0.1366	0.101*	
O3	0.2871 (3)	0.6217 (5)	0.6922 (5)	0.091 (2)	
O4	0.3802 (3)	0.6600 (5)	0.9387 (5)	0.083 (2)	
H4	0.3502	0.6625	0.9002	0.125*	
O5	0.0976 (4)	0.6507 (7)	0.9035 (7)	0.117 (3)	
H5A	0.0871	0.5985	0.9237	0.175*	
C1	−0.2012 (4)	0.6224 (6)	0.0372 (6)	0.052 (2)	
C2	−0.1235 (4)	0.6273 (6)	0.2826 (6)	0.058 (2)	
H2B	−0.1371	0.6369	0.3461	0.070*	
C3	−0.1693 (4)	0.6304 (6)	0.2090 (6)	0.057 (2)	
H3	−0.2124	0.6413	0.2230	0.069*	
C4	−0.1489 (3)	0.6168 (5)	0.1149 (6)	0.0421 (18)	
C5	−0.0846 (4)	0.5984 (5)	0.0988 (6)	0.050 (2)	
H5B	−0.0694	0.5884	0.0362	0.060*	
C6	−0.0435 (4)	0.5950 (5)	0.1764 (6)	0.0484 (19)	
H6	−0.0006	0.5804	0.1649	0.058*	
C7	−0.2105 (4)	0.6256 (6)	−0.2147 (6)	0.054 (2)	
H7	−0.1671	0.6253	−0.2309	0.065*	
C8	−0.2581 (4)	0.6271 (6)	−0.2900 (6)	0.051 (2)	
C9	−0.3244 (4)	0.6246 (6)	−0.2658 (5)	0.051 (2)	
C10	−0.3694 (4)	0.6209 (5)	−0.3420 (6)	0.054 (2)	
C11	−0.3497 (5)	0.6219 (6)	−0.4368 (7)	0.067 (3)	
H11	−0.3798	0.6195	−0.4866	0.081*	
C12	−0.2849 (5)	0.6264 (6)	−0.4578 (6)	0.065 (2)	
C13	−0.2408 (4)	0.6280 (6)	−0.3868 (6)	0.062 (2)	
H13	−0.1974	0.6297	−0.4031	0.074*	
C14	0.2316 (4)	0.6278 (6)	0.7178 (7)	0.059 (2)	
C15	0.0667 (4)	0.6362 (6)	0.5984 (6)	0.062 (2)	
H15	0.0235	0.6397	0.6157	0.074*	
C16	0.1127 (4)	0.6387 (7)	0.6712 (7)	0.069 (3)	
H16	0.1005	0.6465	0.7356	0.082*	
C17	0.1767 (4)	0.6297 (6)	0.6471 (6)	0.054 (2)	
C18	0.1929 (4)	0.6216 (6)	0.5497 (6)	0.062 (2)	
H18	0.2356	0.6160	0.5307	0.074*	
C19	0.1443 (4)	0.6221 (6)	0.4821 (6)	0.059 (2)	
H19	0.1553	0.6174	0.4169	0.071*	
C20	0.2453 (5)	0.6247 (6)	0.9690 (7)	0.069 (3)	
H20	0.2020	0.6160	0.9835	0.083*	
C21	0.2929 (5)	0.6265 (6)	1.0473 (6)	0.062 (3)	
C22	0.3576 (5)	0.6424 (6)	1.0270 (7)	0.064 (2)	
C23	0.3996 (4)	0.6434 (6)	1.1069 (8)	0.068 (3)	
C24	0.3773 (5)	0.6304 (6)	1.2004 (7)	0.072 (3)	
H24	0.4059	0.6305	1.2523	0.086*	
C25	0.3152 (6)	0.6180 (6)	1.2155 (7)	0.076 (3)	
C26	0.2733 (5)	0.6152 (6)	1.1395 (7)	0.069 (3)	
H26	0.2298	0.6051	1.1517	0.083*	
C27	−0.0752 (4)	0.7704 (7)	0.5004 (7)	0.067 (2)	
H27	−0.0956	0.7193	0.5293	0.080*	
C28	−0.0948 (5)	0.8606 (7)	0.5279 (8)	0.075 (3)	
H28	−0.1274	0.8693	0.5731	0.090*	
C29	−0.0642 (5)	0.9364 (9)	0.4857 (7)	0.084 (3)	
H29	−0.0766	0.9976	0.5014	0.101*	
C30	−0.0152 (4)	0.9214 (7)	0.4199 (6)	0.062 (2)	
C31	0.0011 (4)	0.8272 (5)	0.3967 (7)	0.0502 (17)	
C32	0.0493 (4)	0.8083 (6)	0.3272 (6)	0.051 (2)	
C33	0.0833 (5)	0.8831 (6)	0.2830 (6)	0.058 (2)	
C34	0.1290 (4)	0.8613 (7)	0.2135 (7)	0.065 (3)	
H34	0.1528	0.9089	0.1839	0.078*	
C35	0.1386 (4)	0.7700 (8)	0.1893 (7)	0.072 (3)	
H35	0.1679	0.7542	0.1410	0.087*	
C36	0.1037 (4)	0.6990 (7)	0.2380 (6)	0.068 (2)	
H36	0.1110	0.6364	0.2207	0.082*	
C37	0.0213 (5)	0.9959 (7)	0.3775 (8)	0.077 (3)	
H37	0.0130	1.0580	0.3955	0.092*	
C38	0.0686 (5)	0.9776 (7)	0.3105 (8)	0.085 (3)	
H38	0.0912	1.0275	0.2828	0.102*	
C39	0.0539 (9)	0.7183 (13)	0.9363 (15)	0.190 (8)	
H39A	0.0433	0.7058	1.0028	0.286*	
H39B	0.0156	0.7155	0.8976	0.286*	
H39C	0.0727	0.7799	0.9310	0.286*	

### Atomic displacement parameters (Å^2^)

**Table:**

	*U*^11^	*U*^22^	*U*^33^	*U*^12^	*U*^13^	*U*^23^
Co1	0.0473 (6)	0.0645 (6)	0.0411 (5)	0.0011 (5)	−0.0037 (5)	0.0018 (6)
Cl1	0.0679 (14)	0.0748 (14)	0.0607 (13)	0.0178 (11)	−0.0068 (11)	−0.0051 (12)
Cl2	0.0650 (14)	0.0895 (17)	0.0516 (13)	−0.0075 (12)	0.0000 (11)	0.0098 (12)
Cl3	0.0489 (14)	0.148 (3)	0.091 (2)	−0.0081 (15)	−0.0042 (14)	0.0209 (19)
Cl4	0.090 (2)	0.179 (3)	0.0411 (15)	0.0049 (18)	−0.0026 (12)	0.0099 (17)
Cl5	0.0721 (19)	0.146 (3)	0.109 (2)	0.0119 (18)	−0.0259 (17)	−0.036 (2)
Cl6	0.146 (3)	0.131 (3)	0.0541 (16)	0.0219 (19)	−0.0119 (18)	0.0011 (18)
N1	0.040 (4)	0.059 (4)	0.039 (4)	0.008 (3)	−0.004 (3)	−0.003 (3)
N2	0.043 (4)	0.084 (5)	0.040 (4)	0.005 (3)	−0.012 (3)	0.002 (4)
N3	0.049 (4)	0.073 (4)	0.035 (4)	0.006 (3)	−0.008 (3)	−0.003 (3)
N4	0.055 (4)	0.062 (4)	0.042 (4)	0.011 (3)	0.000 (3)	−0.007 (3)
N5	0.061 (5)	0.092 (5)	0.044 (5)	0.005 (4)	−0.013 (4)	−0.005 (4)
N6	0.068 (5)	0.071 (5)	0.046 (5)	0.014 (3)	−0.020 (4)	−0.003 (4)
N7	0.042 (4)	0.086 (5)	0.039 (4)	0.003 (4)	0.001 (3)	−0.008 (4)
N8	0.041 (4)	0.070 (5)	0.039 (4)	0.008 (3)	−0.005 (3)	0.000 (3)
O1	0.050 (4)	0.116 (6)	0.055 (4)	0.016 (4)	−0.002 (3)	−0.015 (4)
O2	0.048 (3)	0.106 (5)	0.049 (4)	0.006 (3)	−0.002 (3)	0.006 (3)
O3	0.049 (4)	0.166 (7)	0.056 (4)	0.010 (4)	−0.015 (3)	−0.018 (4)
O4	0.069 (4)	0.110 (5)	0.070 (5)	0.019 (4)	−0.010 (4)	−0.010 (4)
O5	0.083 (5)	0.166 (8)	0.102 (6)	−0.027 (5)	0.005 (5)	0.039 (6)
C1	0.050 (5)	0.059 (5)	0.046 (5)	0.011 (4)	−0.007 (4)	−0.013 (4)
C2	0.058 (5)	0.086 (6)	0.031 (4)	0.015 (5)	0.009 (4)	0.001 (4)
C3	0.043 (5)	0.088 (6)	0.041 (5)	0.012 (4)	0.002 (4)	−0.017 (4)
C4	0.036 (4)	0.046 (4)	0.044 (4)	0.007 (3)	−0.006 (4)	−0.011 (4)
C5	0.050 (5)	0.059 (5)	0.041 (5)	0.004 (4)	−0.001 (4)	−0.001 (4)
C6	0.042 (4)	0.061 (5)	0.042 (5)	0.001 (4)	0.000 (4)	0.001 (4)
C7	0.052 (5)	0.074 (6)	0.036 (5)	0.005 (4)	−0.014 (4)	0.001 (4)
C8	0.051 (5)	0.063 (6)	0.038 (5)	0.006 (4)	−0.003 (4)	0.002 (4)
C9	0.046 (5)	0.071 (6)	0.036 (5)	−0.001 (4)	−0.005 (4)	0.005 (4)
C10	0.041 (4)	0.063 (5)	0.057 (6)	−0.003 (4)	−0.012 (4)	0.010 (5)
C11	0.061 (6)	0.099 (8)	0.042 (5)	−0.009 (5)	−0.016 (5)	0.001 (5)
C12	0.072 (7)	0.090 (7)	0.034 (5)	−0.010 (5)	−0.007 (4)	0.009 (4)
C13	0.055 (5)	0.090 (7)	0.041 (5)	0.015 (5)	−0.008 (4)	−0.003 (5)
C14	0.052 (6)	0.073 (6)	0.052 (6)	0.002 (5)	−0.017 (4)	0.000 (4)
C15	0.047 (5)	0.096 (7)	0.042 (5)	0.005 (5)	−0.009 (4)	0.000 (5)
C16	0.065 (6)	0.106 (7)	0.034 (5)	0.001 (5)	−0.011 (5)	−0.006 (5)
C17	0.046 (5)	0.064 (5)	0.051 (5)	0.003 (4)	−0.009 (4)	−0.010 (4)
C18	0.045 (5)	0.102 (7)	0.037 (5)	0.000 (5)	−0.009 (4)	−0.006 (5)
C19	0.050 (5)	0.089 (6)	0.039 (5)	−0.009 (5)	−0.009 (4)	0.000 (4)
C20	0.067 (6)	0.083 (7)	0.058 (6)	0.013 (5)	−0.016 (5)	−0.006 (5)
C21	0.083 (8)	0.055 (5)	0.048 (6)	0.015 (5)	−0.024 (5)	−0.010 (4)
C22	0.054 (6)	0.079 (6)	0.059 (6)	0.012 (5)	−0.008 (5)	−0.015 (5)
C23	0.053 (6)	0.070 (6)	0.080 (7)	0.012 (5)	−0.017 (5)	−0.018 (5)
C24	0.085 (8)	0.076 (7)	0.056 (6)	0.016 (6)	−0.023 (5)	−0.018 (5)
C25	0.092 (8)	0.071 (7)	0.065 (7)	0.022 (6)	−0.015 (6)	−0.003 (5)
C26	0.086 (7)	0.064 (6)	0.057 (6)	0.020 (5)	−0.020 (5)	−0.010 (5)
C27	0.062 (6)	0.080 (7)	0.059 (6)	−0.007 (5)	−0.007 (5)	−0.004 (5)
C28	0.046 (6)	0.087 (8)	0.092 (8)	0.012 (5)	0.005 (5)	−0.022 (7)
C29	0.083 (7)	0.103 (9)	0.066 (7)	0.047 (7)	−0.018 (6)	−0.034 (6)
C30	0.054 (5)	0.075 (6)	0.057 (6)	0.011 (5)	−0.018 (4)	−0.010 (5)
C31	0.043 (4)	0.064 (5)	0.044 (4)	−0.002 (4)	−0.009 (4)	−0.008 (5)
C32	0.045 (5)	0.067 (5)	0.040 (5)	−0.002 (4)	−0.013 (4)	−0.001 (4)
C33	0.070 (6)	0.062 (6)	0.043 (5)	−0.006 (5)	−0.018 (4)	0.003 (4)
C34	0.054 (6)	0.077 (7)	0.064 (6)	−0.003 (5)	0.006 (5)	0.022 (5)
C35	0.053 (5)	0.099 (8)	0.064 (6)	0.000 (5)	0.007 (5)	0.017 (6)
C36	0.069 (6)	0.081 (7)	0.055 (6)	0.003 (5)	0.003 (5)	−0.002 (5)
C37	0.093 (7)	0.059 (5)	0.079 (7)	−0.001 (5)	−0.013 (6)	−0.004 (5)
C38	0.106 (9)	0.069 (7)	0.081 (8)	0.000 (6)	−0.018 (7)	0.002 (6)
C39	0.155 (17)	0.21 (2)	0.201 (19)	−0.031 (14)	0.026 (15)	0.014 (17)

### Geometric parameters (Å, °)

**Table:**

Co1—N8	2.170 (7)	C10—C11	1.370 (12)
Co1—N7	2.170 (7)	C11—C12	1.382 (13)
Co1—N1	2.217 (6)	C11—H11	0.9300
Co1—N4	2.235 (7)	C12—C13	1.343 (12)
Co1—Cl1	2.401 (2)	C13—H13	0.9300
Co1—Cl2	2.419 (2)	C14—C17	1.501 (11)
Cl3—C10	1.704 (8)	C15—C16	1.389 (11)
Cl4—C12	1.740 (9)	C15—H15	0.9300
Cl5—C23	1.721 (10)	C16—C17	1.378 (11)
Cl6—C25	1.736 (11)	C16—H16	0.9300
N1—C2	1.321 (9)	C17—C18	1.390 (11)
N1—C6	1.325 (10)	C18—C19	1.376 (11)
N2—C1	1.359 (10)	C18—H18	0.9300
N2—N3	1.368 (8)	C19—H19	0.9300
N2—H2	0.8600	C20—C21	1.465 (12)
N3—C7	1.273 (10)	C20—H20	0.9300
N4—C15	1.327 (10)	C21—C26	1.345 (13)
N4—C19	1.344 (10)	C21—C22	1.393 (13)
N5—C14	1.351 (11)	C22—C23	1.407 (12)
N5—N6	1.389 (9)	C23—C24	1.383 (14)
N5—H5	0.8600	C24—C25	1.321 (14)
N6—C20	1.293 (11)	C24—H24	0.9300
N7—C27	1.309 (11)	C25—C26	1.365 (13)
N7—C31	1.358 (10)	C26—H26	0.9300
N8—C36	1.301 (10)	C27—C28	1.393 (12)
N8—C32	1.360 (10)	C27—H27	0.9300
O1—C1	1.220 (9)	C28—C29	1.377 (14)
O2—C9	1.344 (9)	C28—H28	0.9300
O2—H2A	0.8200	C29—C30	1.382 (12)
O3—C14	1.209 (10)	C29—H29	0.9300
O4—C22	1.329 (11)	C30—C31	1.413 (11)
O4—H4	0.8200	C30—C37	1.426 (12)
O5—C39	1.396 (18)	C31—C32	1.412 (11)
O5—H5A	0.8200	C32—C33	1.412 (11)
C1—C4	1.528 (11)	C33—C34	1.386 (12)
C2—C3	1.392 (11)	C33—C38	1.424 (12)
C2—H2B	0.9300	C34—C35	1.350 (12)
C3—C4	1.378 (10)	C34—H34	0.9300
C3—H3	0.9300	C35—C36	1.411 (12)
C4—C5	1.382 (10)	C35—H35	0.9300
C5—C6	1.370 (10)	C36—H36	0.9300
C5—H5B	0.9300	C37—C38	1.374 (14)
C6—H6	0.9300	C37—H37	0.9300
C7—C8	1.435 (11)	C38—H38	0.9300
C7—H7	0.9300	C39—H39A	0.9600
C8—C13	1.383 (11)	C39—H39B	0.9600
C8—C9	1.418 (11)	C39—H39C	0.9600
C9—C10	1.408 (11)		
			
N8—Co1—N7	76.3 (3)	N5—C14—C17	114.5 (8)
N8—Co1—N1	87.0 (2)	N4—C15—C16	122.7 (8)
N7—Co1—N1	91.8 (2)	N4—C15—H15	118.6
N8—Co1—N4	87.5 (2)	C16—C15—H15	118.6
N7—Co1—N4	88.0 (2)	C17—C16—C15	119.3 (8)
N1—Co1—N4	174.4 (3)	C17—C16—H16	120.3
N8—Co1—Cl1	95.86 (19)	C15—C16—H16	120.3
N7—Co1—Cl1	171.9 (2)	C16—C17—C18	118.3 (7)
N1—Co1—Cl1	90.04 (17)	C16—C17—C14	125.4 (8)
N4—Co1—Cl1	89.40 (17)	C18—C17—C14	116.3 (8)
N8—Co1—Cl2	166.75 (19)	C19—C18—C17	118.5 (8)
N7—Co1—Cl2	90.4 (2)	C19—C18—H18	120.7
N1—Co1—Cl2	93.95 (18)	C17—C18—H18	120.7
N4—Co1—Cl2	91.69 (19)	N4—C19—C18	123.5 (8)
Cl1—Co1—Cl2	97.36 (9)	N4—C19—H19	118.3
C2—N1—C6	117.5 (7)	C18—C19—H19	118.3
C2—N1—Co1	119.3 (5)	N6—C20—C21	120.4 (9)
C6—N1—Co1	123.1 (5)	N6—C20—H20	119.8
C1—N2—N3	116.1 (7)	C21—C20—H20	119.8
C1—N2—H2	122.0	C26—C21—C22	120.1 (9)
N3—N2—H2	122.0	C26—C21—C20	119.3 (10)
C7—N3—N2	118.9 (7)	C22—C21—C20	120.5 (9)
C15—N4—C19	117.6 (7)	O4—C22—C21	123.8 (8)
C15—N4—Co1	121.9 (5)	O4—C22—C23	119.7 (9)
C19—N4—Co1	120.3 (5)	C21—C22—C23	116.4 (9)
C14—N5—N6	116.5 (7)	C24—C23—C22	121.4 (9)
C14—N5—H5	121.7	C24—C23—Cl5	120.1 (8)
N6—N5—H5	121.7	C22—C23—Cl5	118.5 (8)
C20—N6—N5	115.2 (8)	C25—C24—C23	119.5 (9)
C27—N7—C31	117.8 (8)	C25—C24—H24	120.2
C27—N7—Co1	128.3 (6)	C23—C24—H24	120.2
C31—N7—Co1	113.8 (5)	C24—C25—C26	120.5 (11)
C36—N8—C32	117.9 (7)	C24—C25—Cl6	119.0 (8)
C36—N8—Co1	128.2 (6)	C26—C25—Cl6	120.5 (9)
C32—N8—Co1	113.9 (5)	C21—C26—C25	122.0 (11)
C9—O2—H2A	109.5	C21—C26—H26	119.0
C22—O4—H4	109.5	C25—C26—H26	119.0
C39—O5—H5A	109.5	N7—C27—C28	124.7 (9)
O1—C1—N2	122.5 (7)	N7—C27—H27	117.7
O1—C1—C4	121.5 (7)	C28—C27—H27	117.7
N2—C1—C4	116.0 (7)	C29—C28—C27	117.7 (9)
N1—C2—C3	123.4 (8)	C29—C28—H28	121.2
N1—C2—H2B	118.3	C27—C28—H28	121.2
C3—C2—H2B	118.3	C28—C29—C30	119.9 (9)
C4—C3—C2	118.2 (7)	C28—C29—H29	120.0
C4—C3—H3	120.9	C30—C29—H29	120.0
C2—C3—H3	120.9	C29—C30—C31	118.1 (9)
C3—C4—C5	118.4 (7)	C29—C30—C37	123.3 (10)
C3—C4—C1	115.8 (7)	C31—C30—C37	118.5 (8)
C5—C4—C1	125.8 (7)	N7—C31—C32	118.0 (7)
C6—C5—C4	119.0 (7)	N7—C31—C30	121.8 (8)
C6—C5—H5B	120.5	C32—C31—C30	120.2 (8)
C4—C5—H5B	120.5	N8—C32—C31	117.7 (7)
N1—C6—C5	123.5 (7)	N8—C32—C33	121.8 (8)
N1—C6—H6	118.3	C31—C32—C33	120.4 (8)
C5—C6—H6	118.3	C34—C33—C32	118.4 (9)
N3—C7—C8	120.6 (8)	C34—C33—C38	122.8 (9)
N3—C7—H7	119.7	C32—C33—C38	118.9 (9)
C8—C7—H7	119.7	C35—C34—C33	119.1 (9)
C13—C8—C9	118.8 (7)	C35—C34—H34	120.5
C13—C8—C7	121.2 (8)	C33—C34—H34	120.5
C9—C8—C7	120.0 (7)	C34—C35—C36	119.3 (9)
O2—C9—C10	118.6 (7)	C34—C35—H35	120.3
O2—C9—C8	123.3 (7)	C36—C35—H35	120.3
C10—C9—C8	118.1 (7)	N8—C36—C35	123.4 (9)
C11—C10—C9	120.9 (8)	N8—C36—H36	118.3
C11—C10—Cl3	119.3 (7)	C35—C36—H36	118.3
C9—C10—Cl3	119.8 (7)	C38—C37—C30	121.2 (9)
C10—C11—C12	119.5 (8)	C38—C37—H37	119.4
C10—C11—H11	120.3	C30—C37—H37	119.4
C12—C11—H11	120.3	C37—C38—C33	120.6 (10)
C13—C12—C11	121.0 (8)	C37—C38—H38	119.7
C13—C12—Cl4	121.7 (8)	C33—C38—H38	119.7
C11—C12—Cl4	117.4 (7)	O5—C39—H39A	109.5
C12—C13—C8	121.7 (8)	O5—C39—H39B	109.5
C12—C13—H13	119.1	H39A—C39—H39B	109.5
C8—C13—H13	119.1	O5—C39—H39C	109.5
O3—C14—N5	123.0 (8)	H39A—C39—H39C	109.5
O3—C14—C17	122.4 (8)	H39B—C39—H39C	109.5
			
N8—Co1—N1—C2	−126.7 (6)	C9—C8—C13—C12	−0.1 (14)
N7—Co1—N1—C2	−50.5 (6)	C7—C8—C13—C12	177.8 (8)
N4—Co1—N1—C2	−138 (2)	N6—N5—C14—O3	4.1 (14)
Cl1—Co1—N1—C2	137.5 (6)	N6—N5—C14—C17	−177.0 (7)
Cl2—Co1—N1—C2	40.1 (6)	C19—N4—C15—C16	1.3 (13)
N8—Co1—N1—C6	51.9 (6)	Co1—N4—C15—C16	176.3 (7)
N7—Co1—N1—C6	128.1 (6)	N4—C15—C16—C17	−2.7 (14)
N4—Co1—N1—C6	40 (3)	C15—C16—C17—C18	2.2 (13)
Cl1—Co1—N1—C6	−43.9 (6)	C15—C16—C17—C14	−177.8 (8)
Cl2—Co1—N1—C6	−141.3 (6)	O3—C14—C17—C16	−178.4 (9)
C1—N2—N3—C7	178.4 (7)	N5—C14—C17—C16	2.6 (13)
N8—Co1—N4—C15	136.9 (7)	O3—C14—C17—C18	1.6 (13)
N7—Co1—N4—C15	60.5 (7)	N5—C14—C17—C18	−177.4 (8)
N1—Co1—N4—C15	148 (2)	C16—C17—C18—C19	−0.6 (13)
Cl1—Co1—N4—C15	−127.2 (6)	C14—C17—C18—C19	179.4 (8)
Cl2—Co1—N4—C15	−29.9 (6)	C15—N4—C19—C18	0.4 (13)
N8—Co1—N4—C19	−48.3 (6)	Co1—N4—C19—C18	−174.7 (7)
N7—Co1—N4—C19	−124.7 (6)	C17—C18—C19—N4	−0.7 (14)
N1—Co1—N4—C19	−37 (3)	N5—N6—C20—C21	179.9 (7)
Cl1—Co1—N4—C19	47.6 (6)	N6—C20—C21—C26	−179.7 (9)
Cl2—Co1—N4—C19	145.0 (6)	N6—C20—C21—C22	2.0 (13)
C14—N5—N6—C20	−170.0 (8)	C26—C21—C22—O4	−175.7 (9)
N8—Co1—N7—C27	179.8 (8)	C20—C21—C22—O4	2.5 (14)
N1—Co1—N7—C27	93.3 (7)	C26—C21—C22—C23	1.5 (13)
N4—Co1—N7—C27	−92.3 (7)	C20—C21—C22—C23	179.7 (8)
Cl1—Co1—N7—C27	−164.0 (10)	O4—C22—C23—C24	176.4 (9)
Cl2—Co1—N7—C27	−0.6 (7)	C21—C22—C23—C24	−0.8 (13)
N8—Co1—N7—C31	−3.9 (5)	O4—C22—C23—Cl5	−4.0 (12)
N1—Co1—N7—C31	−90.4 (5)	C21—C22—C23—Cl5	178.8 (6)
N4—Co1—N7—C31	84.0 (6)	C22—C23—C24—C25	−0.8 (14)
Cl1—Co1—N7—C31	12.3 (17)	Cl5—C23—C24—C25	179.6 (7)
Cl2—Co1—N7—C31	175.6 (5)	C23—C24—C25—C26	1.9 (15)
N7—Co1—N8—C36	−175.4 (7)	C23—C24—C25—Cl6	−176.6 (7)
N1—Co1—N8—C36	−82.8 (7)	C22—C21—C26—C25	−0.5 (13)
N4—Co1—N8—C36	96.1 (7)	C20—C21—C26—C25	−178.7 (8)
Cl1—Co1—N8—C36	6.9 (7)	C24—C25—C26—C21	−1.2 (14)
Cl2—Co1—N8—C36	−177.2 (6)	Cl6—C25—C26—C21	177.2 (7)
N7—Co1—N8—C32	3.5 (5)	C31—N7—C27—C28	2.1 (13)
N1—Co1—N8—C32	96.0 (5)	Co1—N7—C27—C28	178.3 (7)
N4—Co1—N8—C32	−85.1 (5)	N7—C27—C28—C29	−0.6 (15)
Cl1—Co1—N8—C32	−174.2 (5)	C27—C28—C29—C30	−1.2 (14)
Cl2—Co1—N8—C32	1.6 (11)	C28—C29—C30—C31	1.3 (13)
N3—N2—C1—O1	−3.2 (11)	C28—C29—C30—C37	−176.2 (9)
N3—N2—C1—C4	179.1 (6)	C27—N7—C31—C32	−179.4 (7)
C6—N1—C2—C3	−2.0 (12)	Co1—N7—C31—C32	3.9 (9)
Co1—N1—C2—C3	176.7 (7)	C27—N7—C31—C30	−2.0 (12)
N1—C2—C3—C4	−0.5 (14)	Co1—N7—C31—C30	−178.7 (6)
C2—C3—C4—C5	1.7 (13)	C29—C30—C31—N7	0.3 (12)
C2—C3—C4—C1	−179.2 (7)	C37—C30—C31—N7	177.9 (8)
O1—C1—C4—C3	−5.7 (12)	C29—C30—C31—C32	177.6 (7)
N2—C1—C4—C3	172.0 (8)	C37—C30—C31—C32	−4.8 (12)
O1—C1—C4—C5	173.3 (8)	C36—N8—C32—C31	176.3 (7)
N2—C1—C4—C5	−9.0 (11)	Co1—N8—C32—C31	−2.7 (9)
C3—C4—C5—C6	−0.5 (12)	C36—N8—C32—C33	−4.4 (11)
C1—C4—C5—C6	−179.5 (7)	Co1—N8—C32—C33	176.6 (6)
C2—N1—C6—C5	3.4 (12)	N7—C31—C32—N8	−0.9 (11)
Co1—N1—C6—C5	−175.3 (6)	C30—C31—C32—N8	−178.3 (7)
C4—C5—C6—N1	−2.1 (12)	N7—C31—C32—C33	179.8 (7)
N2—N3—C7—C8	178.5 (7)	C30—C31—C32—C33	2.4 (12)
N3—C7—C8—C13	−179.8 (9)	N8—C32—C33—C34	2.3 (12)
N3—C7—C8—C9	−1.9 (12)	C31—C32—C33—C34	−178.4 (8)
C13—C8—C9—O2	−179.8 (8)	N8—C32—C33—C38	−178.6 (8)
C7—C8—C9—O2	2.3 (12)	C31—C32—C33—C38	0.7 (12)
C13—C8—C9—C10	1.5 (12)	C32—C33—C34—C35	1.2 (13)
C7—C8—C9—C10	−176.5 (7)	C38—C33—C34—C35	−177.8 (9)
O2—C9—C10—C11	179.7 (8)	C33—C34—C35—C36	−2.5 (14)
C8—C9—C10—C11	−1.5 (12)	C32—N8—C36—C35	3.1 (12)
O2—C9—C10—Cl3	0.1 (11)	Co1—N8—C36—C35	−178.1 (6)
C8—C9—C10—Cl3	178.9 (6)	C34—C35—C36—N8	0.3 (14)
C9—C10—C11—C12	0.2 (13)	C29—C30—C37—C38	−178.3 (9)
Cl3—C10—C11—C12	179.7 (7)	C31—C30—C37—C38	4.3 (14)
C10—C11—C12—C13	1.3 (14)	C30—C37—C38—C33	−1.3 (15)
C10—C11—C12—Cl4	−178.3 (7)	C34—C33—C38—C37	177.8 (9)
C11—C12—C13—C8	−1.3 (15)	C32—C33—C38—C37	−1.2 (14)
Cl4—C12—C13—C8	178.3 (7)		

### Hydrogen-bond geometry (Å, °)

**Table:**

*D*—H···*A*	*D*—H	H···*A*	*D*···*A*	*D*—H···*A*
N2—H2···Cl1^i^	0.86	2.56	3.280 (7)	142
N5—H5···O5	0.86	1.91	2.739 (11)	162
O2—H2A···N3	0.82	1.85	2.562 (8)	145
O4—H4···N6	0.82	1.88	2.592 (9)	145
O5—H5A···Cl2^ii^	0.82	2.24	3.052 (9)	171

Symmetry codes: (i) −*x*, −*y*+1, *z*−1/2; (ii) −*x*, −*y*+1, *z*+1/2.
